# Society Meetings

**Published:** 1905-04

**Authors:** 


### SOCIETY MEETINGS.

THE ESCULAPIAN CLUB held its regular monthly meeting at the Hotel Touraine, Thursday evening, March 16, 1905, at 8.30. The host was Dr. George B. Stocker, and the subject of the paper was Cerebral abscess, presented by Dr. J. G. Ernest.

---

THE Buffalo Academy of Medicine held meetings during the months of February and March, 1905, as follows:

*Section on Obstetrics and Gynecology.*—Tuesday evening, February 28. Program: (*a*) Gonorrhea and the woman, S. Y. Howell; (*b*) The need of a medical library in Buffalo, W. C. Krauss.

*Section on Surgery.*—Tuesday evening, March 7. Program: (*a*) Treatment of hallux valgus, Vertner Kenerson; (*b*) Movable kidney, A. L. Benedict.

*Section on Medicine.*—Tuesday evening, March 14. Program: (*a*) Some of the newer problems in diabetes mellitus, Elliott T. Joslin, Boston; (*b*) The care of premature infants, Dewitt H. Sherman.

*Section on Pathology.*—Tuesday evening, March 21. Program: (1) A discussion of humidity in artificial heating and its relation to disease, B. F. Herr, Millersville, Pa.; (2) Anatomy in its relation to the explanation of certain pains, J. A. Gibson; (3) Case of spleno-myelogenic leukemia, with patient, Norman L. Burnham; (4) Specimen of ectopic gestation, Thomas H. McKee; (5) Model of the male perineum, David E. Wheeler; (6) Model of the larynx, Herman K. De Groat.

*Section on Obstetrics and Gynecology.*—Tuesday evening, March 28. Nominations of officers for the ensuing year were made at this meeting. Program: (*a*) Cesarean section, Charles E. Congdon; (*b*) Report of a case of Cesarean section, Grosvenor R. Trowbridge.

THE Erie County Medical Association held its annual meeting at the University Club, Monday evening, March 13, 1905. Program: (1) Presentation of cases and specimens; (2) Experimental work to devise an operation which would simplify the treatment of advanced cases of lateral curvature of the spine, Prescott LeBreton; (3) Treatment of acute otitis media, W. Scott Renner; (4) The busy practitioner from a business point of view, Albert J. Colton.

---

THE Physicians' Club of Buffalo held its regular monthly meeting Friday evening, March 3, 1905, at the residence of Dr. J. J. Finerty, the members and invited guests numbering about sixty. Dr. William C. Callahan read a paper entitled, Pneumoconiosis, which was discussed by D. J. Constantine, C. E. Congdon, William G. Ring, Thomas B. Carpenter, J. Henry Dowd, S. Y. Howell and others. The election of officers for the ensuing year resulted as follows: president, James S. Porter; vice-president, D. J. Constantine; secretary-treasurer, William G. Ring.

